# Survey of Early Complications of Primary Skin Graft and Secondary Skin Graft (Delayed) Surgery after Resection of Burnwaste in Hospitalized Burn Patients

**DOI:** 10.5539/gjhs.v6n7p98

**Published:** 2014-09-18

**Authors:** A. Enshaei, N. Masoudi

**Affiliations:** 1 Urmia University of Medical Sciences and Health Services, Urmia, Iran

**Keywords:** burn waste, primary skin graft, secondary skin graft, complications

## Abstract

**Introduction:**

Burning is the second most common cause of home injuries in Iran that is often the cause of conflicts between children and young adults. Burning can lead to early and late complications that scar and contracture are the most common. Burn waste treatment is done by two methods: excision and then skin graft after the formation of granulation tissue; and excision and graft simultaneously that in this study, these two methods are compared.

**Methods:**

This was performed as a quasi-experimental analysis and retrospective study on all patients who were hospitalized for burn scar. All patients who have associated with weak eningimmune diseases such as diabetes, acquired immunodeficiency or congenital, taking steroids and patients undergoing chemotherapy etc. are excluded. The method of grafting in patients is primary graft procedure that was compared with patients who are treated using secondary graft. Data collected through review of patients’ hospital and clinic chart.

**Results:**

The mean burn percentage in the primary repair group was 14.4% and in the delayed repair group was 16.6%, respectively. The incidence of hematoma in both groups was zero. Skin necrosis and graft rejection and infection in the primary repair group was in 3.7% of patients and in the delayed repair group was in 1.2% of cases (P=0.5)

**Conclusion:**

Based on the findings of this study, no difference was observed between the two methods of excision and primary graft with delayed graft in the incidence of graft rejection. Due to the shorter treatment of primary graft and patient satisfaction and also according to the findings of this study excision and primary graft method seems appropriate method for treating old waste burning.

## 1. Introduction

Thermal burns and associated injuries are among the most common causes of death and disability. The introduction of specialized burn centers for treating has been a major step in treating these patients, as for the treatment, a medical group consists of several specialties, including surgical, rehabilitation, psychiatry, nursing and nutritionist etc. is required (Kushyar et al., 2004; Derakhshan et al., 1998; Studenejad & Janghorbani, 1995, Khosravi & Amuzeghar, 1999; Shekrvssh, 1996; Atari & Mohammadreza, 1997; Ross, 2004; Steven & Wolf, 2004)

### 1.1 Epidemiology

In the United States, about 1.1 million people are in need of medical intervention are burned annually. Of these, 45,000 require hospitalization and 4,500 will die (James & David, 2005).

The quality of burn treatment is not clear only by survival of the patient, but the long-term function and the patient’s appearance is also important (Jang, Teng, Ojo, & Genden, 2013). The goal of treatment in a burned patient is that the patient become quite good, function returns, and has a good appearance (James & David, 2005).

As in other cases of trauma, burning is also seen more in younger people. Less than 8 years of age often hot liquids and in older ages heat and fire are the causes of burning. Chemical and electrical burns are more related to jobs (James & David, 2005).

Burning is the second most common cause of home injuries in Iran that is often the cause of conflicts between children and young adults. (Khosravi et al., 1999; Atari & Mohammadreza, 1997) Boys are burned more than girls that orderly, hot liquids and flames are the common causes (Studenejad & Janghorbani, 1995; Shekrvssh, 1996). Burning can lead to early and late complications that scar and contracture is the most common (Derakhshan, 1998).

Burn scar can cause temporary or permanent disability is a person who has various social and economic consequences. So considering the treatment of this group of patients and increase the quality of this issue will be followed by many benefits (Sahin, 2014).

In some cases burn scar is deep and involving articular surfaces, genitals, face, etc. that cause sorgan dysfunction and its beauty (Steven & Wolf, 2004; James & David, 2005).

Skin scar is often created in cases of deep burns of grade II and grade III and IV that will usually require skin graft.

Another complication is keloid, which is created due to increased inflammatory process and innate talent of patient, so that unlike hypertrophic scars, keloid goes beyond the scope of scar and is larger and is not self-limiting (James & David, 2005).

The scars, in terms of both aesthetic and functional, need excision surgery that usually injury area and tissue defect are treated by skin grafts or muscle and skin flaps.

One goal of treatment is to restore function and normal appearance of involved organ. To achieve this, several treatments are usually needed, which requires a long time.

Burn scars treated by skin grafts typically done in two stages: the first stage is scar excision and releasing contractures and the next step is skin graft. Depending on the location and the organ involved, the time interval between scar excision and restoration is between 1-3 weeks variable (Ross, 2004).

In this time interval patient needs to replace daily dressings that are very painful, besides in this period patient would have no social function (Ross, 2004; Steven & Wolf, 2004; James & David, 2005; John et al., n.d.).

### 1.2 Reasons for Rejection of Skin Graft

* Unsuitable platform (insufficient blood supply)

*Moving and displacement

*Technical error, such as graft one pithelium tissue or a too thick graft or too thin

*Improper maintenance and storage

*Hematoma

*Infection

What is important in determining the outcome of skin grafts is graft perfusion and nutrition (Ross, 2004; Steven & Wolf, 2004); this time delay is usually to prepare a proper blood supply to the graft, but this time will cause negative effects such as the colonization of organisms in the tissue which will increase infection and graft rejection, in addition, in many cases the same basic platform is also appropriate condition for grafting.

In this study we tried to compare the patients whose burning waste is restored in one step (along with excision of wastes, skin grafts) with patients who are restored in two steps (first excision of wastes after the formation of granulation tissue, skin grafts).

Removing this to me will prevent painful dressing changes; patients return to their works and daily activities as well as reduce the cost of treatment.

Given that burning is created in the majority of young people in society that are parts of efficient members of present and future of society, is the necessity to properly treat the disease.Thus reducing the duration of treatment and absence from work will be of great interests to society.

The aim of this study was to compare the early complications of primary skin grafts and secondary skin grafts (delayed) after resection of burning waste in burn patients hospitalized in Imam Khomeini Hospital, Urmia in 2010.

## 2. Methods

This study was carried out in Imam Khomeini Hospital of Urmia on patients admitted to this center with detection of burn scar.

In this study all the patients, who have been hospitalized for scar in 2010, at this center were studied. The method of grafting in patients is primary graft procedure hat was compared with patients who were treated using secondary grafts.

This was performed as a quasi-experimental analysis and retrospective study on all patients who were hospitalized for burn scar. All patients who have associated with weakening immune diseases such as diabetes, acquired immunodeficiency or congenital, taking steroids and patients undergoing chemotherapy etc. are excluded.

In this study all patients, whose burn waste shave been treated with primary graft were compared with patients who were treated using secondary grafts. In this study, type of waste and its location, extent and size of them are not considered in the sample selection. There were no age limit and all patients who have been hospitalized with burn scar detection participated in the study.

By studying the hospital records of patients and their clinical records data were collected and entered into the questionnaire.

For analysis of qualitative data and evaluation of the differences between variables Chi-Square qualitative Statistical method was used. Independent T-Test was used for the analysis of quantitative data. A significant amount of range (A error) equivalent to 0.05 was considered. SPSS 15 computer program was used for data analysis (Dalfard, 2014).

## 3. Results

All medical records of patients that were admitted to Imam Khomeini Hospital Urmia, in 2010, with burn scar were studied. Records of 121 patients, who due to old burn scar had undergone repair surgery, were eligible to participate in the study.

During 2010 a total of 121 patients who had undergone burn repair surgery, 94 cases had delayed healing and 27 patients had primary repair and excision. The mean burn percentage in the primary repair group was 14.4% and in the delayed repair group was 16.6%, respectively (Table 1).

**Table 1:** Mean burn percentage in primary repair group and delayed repair group

	Type of surgery	Number	Mean	SD
Burn percentage	Primary	27	370.14	2274.6
	Delayed	94	896.16	6078.6

The mean age at primary graft group was 28.3 years, and in secondary graft group was 24 years (Table 2).

**Table 2:** The mean age of patients in the primary repair group and delayed repair group

	Type of surgery	Number	Mean	SD
Age	Primary	27	296.28	6212.12
	Delayed	94	979.23	9946.11

In primary graft group 59.3% was female and 40.7% was male and in secondary graft group 57.4% was female and 42.6 were male. The most common location of burn repair in primary method was head and neck (50%). In the secondary repair no particular site in the body was more common.

Listed in patients’ records files, all patients had no history of a particular disease and there was no positive point in cases in favor of underlying disease.

The incidence of hematoma was zero in both groups, and no cases of hematoma have been reported.

Skin necrosis and graft rejection in the primary repair group was in 3.7% of patients and in the delayed repair group was in 2.1% of the cases and the incidence of graft infection was 3.7% in the primary repair group and 2.1% in the delayed repair group.

According to the above two cases (infection and graft rejection) seems all the rejection were due to infection. Grafted skin pigmentation was seen to varying degrees in all cases, but in this case because of defect records and short follow-up time commenting and making conclusions in this case would be invalid.

## 4. Discussion

Burning is the second most common cause of home injuries in Iran that is often the cause of conflicts between children and young adults (Khosravi, 2003; Atari, 1997). Boys are burned more than girls that orderly, hot liquids and flames are the common causes (Studenejad & Janghorbani, 1995; Shekrvssh, 1996). Burning can lead to early and late complications that scar and contracture are the most common.

Skin scar is often created in cases of deep burns of grade II and grade III and IV that will usually requires kin graft.

One goal of treatment is to restore function and normal appearance of involved burn organs. Often to achieve this, multiple treatments are needed, which requires a longtime.

Burn scars treated by skin grafts typically done in two stages: the first stage is scar excision and releasing contractures and the next step is skin graft. Depending on the location and the organ involved, the time interval between scar excision and restoration is between 1-3 weeks variable (Ross, 2004).

In this time interval patient needs to replace daily dressings that are very painful, besides in this period patient would have no social function.

What is important in determining the outcome of skin grafts is graft perfusion and nutrition (Ross 2004; Steven & Wolf, 2004); this time delay is usually to prepare a proper blood supply to the graft, but this time will cause negative effects such as the colonization of organisms in the tissue which will increase infection and graft rejection, in addition, in many cases the same basic platform is also appropriate condition for grafting.

One of the complications of burning is its social pathology. Absence from work, early failure of efficient force, and multiple traumas inflicted on the patient and his family, enormous medical costs etc. are all the reasons that require the necessity of considering to treatment of these patients. What is obvious, the long time required for full recovery can be the justification for many of these injuries.

To obtain a better recovery of burn scars, multiple treatments are applied, such as different skin grafts, diverse skin or skin–muscle flaps or the use of different types of prostheses (Steven & Wolf, 2004).

According to major shortcomings in the source graft in burn patients, operation with a high chance of success will be of two-fold importance in these patients (Ross, 2004).

The most common performed repair surgery is skin graft in these patients. The main problem with this method is the long time required to achieve full recovery.

In this study, we tried to compare two methods of delayed healing of burn wastes and primary repair simultaneously with scar excision.

As shown in this study, the incidence of graft rejection, in two primary graft groups following excision and delayed graft after granulation tissue formation was equal and were not significantly different.

Hematoma and infection that are the main reasons for the rejection were similar in both groups.

The color change that is a graft complication is primarily in the form of pigmentation, which was observed in both groups.

What clearly appears was one step operation and avoidance of painful dressing changes.

According to our findings, it is appears that we can excision old burn scars, keloid tissue, joint contractures, or hypertrophic burn scars in a single stage operation and at the same time also attempt to graft the half-thickness or full thickness skin.

In the studies that had been done in the past, similar results were obtained (Pham, Hanley, & Palmieri, 2001; Mita, Prabodh, & Thomas, 2004). However, these studies have been done mainly for acute burns wastes that necessitate the need for a study of old burn wastes.

**Suggestions:**

*Conduct a study with the same title but with longer follow-up period to investigate late effects of skin grafts that may occur over several years.

*Conduct a study to comparative examination of skin contractures in primary and delayed grafts.

*Conduct a study aimed to compare treatment costs of two methods of primary and delayed graft until the proper recovery time.

*Conduct a prospective study to determine the requirements for substrate tissue for primary graft.
